# Disparities in Healthcare Utilisation Rates for Aboriginal and Non-Aboriginal Albertan Residents, 1997–2006: A Population Database Study

**DOI:** 10.1371/journal.pone.0048355

**Published:** 2012-11-12

**Authors:** Helen Chung, Ming Ye, Chris Hanson, Oluwaseun Oladokun, Michael J. Campbell, Gordon Kramer, Ordan J. Lehmann

**Affiliations:** 1 Department of Ophthalmology, University of Alberta, Edmonton, Alberta, Canada; 2 Department of Public Health Sciences, University of Alberta, Edmonton, Alberta, Canada; 3 Health Services Research, ScHARR, University of Sheffield, Sheffield, United Kingdom; 4 Funding and Methodologies, Capital Health Region, Edmonton, Alberta, Canada; 5 Department of Medical Genetics, University of Alberta, Edmonton, Alberta, Canada; UNAIDS, Switzerland

## Abstract

**Background:**

It is widely recognised that significant discrepancies exist between the health of indigenous and non-indigenous populations. Whilst the reasons are incompletely defined, one potential cause is that indigenous communities do not access healthcare to the same extent. We investigated healthcare utilisation rates in the Canadian Aboriginal population to elucidate the contribution of this fundamental social determinant for health to such disparities.

**Methods:**

Healthcare utilisation data over a nine-year period were analysed for a cohort of nearly two million individuals to determine the rates at which Aboriginal and non-Aboriginal populations utilised two specialties (Cardiology and Ophthalmology) in Alberta, Canada. Unadjusted and adjusted healthcare utilisation rates obtained by mixed linear and Poisson regressions, respectively, were compared amongst three population groups - federally registered Aboriginals, individuals receiving welfare, and other Albertans.

**Results:**

Healthcare utilisation rates for Aboriginals were substantially lower than those of non-Aboriginals and welfare recipients at each time point and subspecialty studied [e.g. During 2005/06, unadjusted Cardiology utilisation rates were 0.28% (Aboriginal, n = 97,080), 0.93% (non-Aboriginal, n = 1,720,041) and 1.37% (Welfare, n = 52,514), p = <0.001]. The age distribution of the Aboriginal population was markedly different [2.7%≥65 years of age, non-Aboriginal 10.7%], and comparable utilisation rates were obtained after adjustment for fiscal year and estimated life expectancy [Cardiology: Incidence Rate Ratio 0.66, Ophthalmology: IRR 0.85].

**Discussion:**

The analysis revealed that Aboriginal people utilised subspecialty healthcare at a consistently lower rate than either comparatively economically disadvantaged groups or the general population. Notably, the differences were relatively invariant between the major provincial centres and over a nine year period. Addressing the causes of these discrepancies is essential for reducing marked health disparities, and so improving the health of Aboriginal people.

## Introduction

The significant disparity between the health of Aboriginal and non-Aboriginal populations is a worldwide phenomenon [1]–[8]. Multiple causes have been suggested including socio-economic inequality [4]–[5], [8]–[15], geographic isolation from healthcare facilities [3], [5], [11], and socio-cultural variation among different ethnic groups [4]–[5], [9], [16]–[18]. In Canada, there are comparable findings of a disproportionate disease burden, with Aboriginal populations also demonstrating higher mortality rates [4]–[5], [11], [13]–[14] and a 6.6 year shorter life expectancy than that of the general population [19]. Despite recent improvements, the discrepancies in life expectancy and infant mortality remain pronounced [20].

The objective of this study was to determine whether Aboriginal status was associated with lower healthcare access rates. Since this key social determinant of health [21] represents the first step in receiving treatment, discrepancies at this stage would be expected to manifest in subsequent morbidity, potentially for a range of diseases. However, since access to healthcare is challenging to quantify, as a surrogate measure, the province's hospital utilisation and health care insurance records were analyzed to investigate if healthcare utilisation differed between Aboriginals and non-Aboriginals. It was anticipated that such methodology would provide a novel approach for measuring provincial healthcare utilisation, reflecting both clinical and societal factors. Overall, this study provides data supporting the hypothesis that Aboriginal peoples utilise healthcare at lower rates than the general population and reveals ways in which this issue can be addressed.

## Methods

### Population and Utilisation Data

Computerised databases of healthcare utilisation and provincial health care insurance information for the province of Alberta's 3.29 million residents [22], were available. These comprised data on each patient's: age range, location and timing of the clinical episode, subspecialty service accessed, distance from the visited healthcare facility and socioeconomic status.

The utilisation records were interrogated for clinical episodes in Cardiology and Ophthalmology, subspecialties selected because they treat medical and surgical patients of all ages, with contrasting emphases on morbidity and mortality. The latter is exemplified by cardiac disease being the second leading cause of death in Canada [23] and in the Aboriginal population [24]. Notably, these two sub-specialties encompass treatment of major complications of diabetes (retinopathy and maculopathy; and ischemic heart disease) [25]–[26], a disorder substantially more prevalent in Canadian Aboriginal [27] and indigenous populations worldwide [28]. Accordingly, Cardiology and Ophthalmology were considered representative of the range of clinical disciplines with their delivery primarily in the province's major cities (Edmonton and Calgary), representing an additional advantage. Collected data, which included both inpatient and outpatient episodes, had been anonymised and then pooled into 5 year age cohorts, prior to being released for analysis. Individuals whose healthcare is provided by the federal government [Royal Canadian Mounted Police, Armed Forces and inmates at federal penitentiaries] as well as recent arrivals from other provinces are included in the utilisation but not the population data, whilst the converse applies to Albertans temporarily living outside the province and those with valid work or student visas.

The insurance records were used to stratify individuals according to whether they were federally registered Aboriginals, individuals receiving welfare (who were not federally registered Aboriginals), or other Albertan residents. These three groups comprise substantial populations (77 000–97 000; 52 000–61 000; and 1.49–1.72 million individuals, respectively) within the province of Alberta for the period studied (1997–2006), and their composition is tightly defined. Federally registered Aboriginal status is specified by an act of Parliament [the Indian Act of 1876] [29] with such individuals comprising 53% of Canadian Aboriginal people [30] and receiving limited benefits under treaties signed by British and, from 1867 Canadian, governments. The population receiving welfare is similarly well delineated, receiving government-funded Income Support to meet basic needs, up to age 65.

To determine whether geographical distance from the main subspecialty centres (Edmonton and Calgary) influenced healthcare utilisation, healthcare utilisation rates were calculated using postal codes for geographic regions encompassing all forty-eight Albertan First Nation reserves and Métis settlements. Rates were calculated for each region, based on the number of instances a subspecialty was utilised each fiscal year (1997–2006). Comparable analyses were undertaken for the cities of Edmonton and Calgary that each have very substantial Aboriginal populations (52 100 and 26 575, respectively) [31].

### Healthcare Utilisation Rate Calculation and Statistical Analysis

Healthcare utilisation rates were calculated using two different approaches. Unadjusted rates for each population group per fiscal year, were derived from the ratio of the group's number of subspecialty visits in Edmonton or Calgary to the total number of individuals in that group. For instance, the 2005/2006 Aboriginal Cardiology utilisation rate for Calgary was determined by dividing the number of visits by the total number of Albertan Aboriginals listed in the insurance records of that fiscal year. A linear mixed model for longitudinal data [32]–[33] was used to compare utilisation rates between Aboriginal, welfare-recipient, and non-Aboriginal groups over nine years.

**Figure 1:**
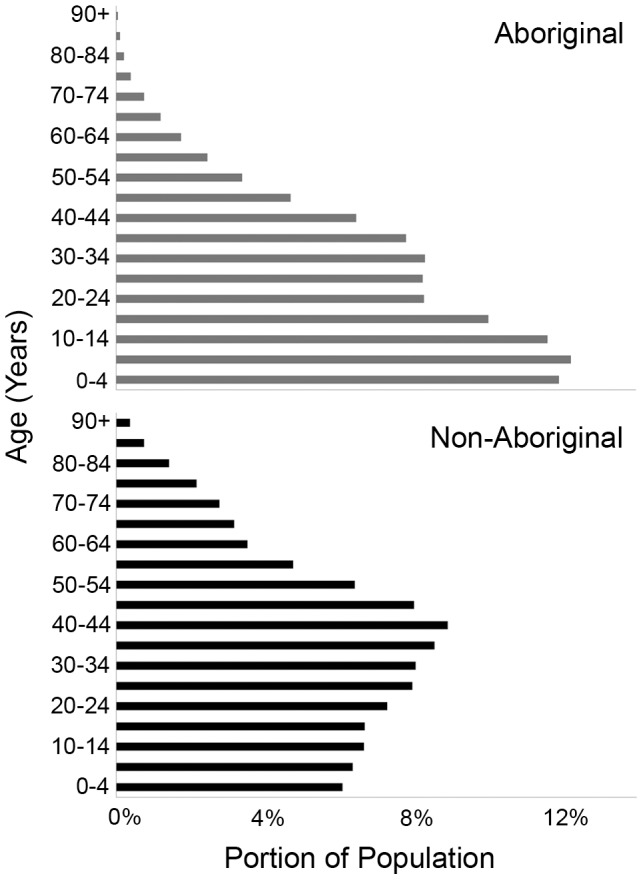
Average age distributions of Albertan Aboriginal and non-Aboriginal populations from 1997–2006. Note the profound differences in longevity and the smaller proportions of Aboriginals over 65 years of age.

Adjusted utilisation rates were calculated using two different methodologies to address potential confounding by the shorter life span of Aboriginal peoples [19]. First, an indirect standardisation was carried out using Poisson regression [34] (STATA 11, StataCorp, TX, USA) [35]. This involved including age groups (as 5 year categories), fiscal year, and ethnicity/welfare in a model with number of visits as the dependent variable and the log of population size as an offset. This produced an Incidence Rate Ratio (IRR) - the ratio of the utilisation rate for the Aboriginals or Welfare-receiving population standardised for age, relative to the general population. In parallel, to address their 6.6 year reduced longevity [19], rather than compare a 70 year old Aboriginal (∼3 year life expectancy) with a 70 year old Albertan (∼10 year life expectancy), individuals of comparable life expectancy were evaluated. This was achieved by comparing Aboriginals aged x with non-Aboriginals aged x+5 years. This conservative correction accords with this study's demographic data (Figure 1), is less than the reduction in Aboriginal life expectancy estimated by Indian and Northern Affairs Canada [19], and reflects the partitioning of our data into 5 year age cohorts. The IRR data derived by correcting for age, and that provided by adjusting for life expectancy, are provided in Table 1. Finally, additional Poisson regression analyses were undertaken to determine if any interaction existed between age-corrected health care utilisation and ethnicity (Table S1).

**Table 1:** Healthcare utilisation rates with adjustment of fiscal year and either chronological age or estimated life expectancy.

Subspecialty	Population Group (chronological age or life expectancy correction)	Incidence Rate Ratio (95% CI)	p-value
Cardiology	Non-Aboriginal	1	
	Welfare	2.31 (2.14 to 2.48)	<0.0001
	Aboriginal (chronological age)	0.97 (0.89 to 1.04)	0.362
	Aboriginal (life expectancy)	0.66 (0.62 to 0.71)	<0.0001
Ophthalmology	Non-Aboriginal	1	
	Welfare	1.81 (1.71 to 1.92)	<0.0001
	Aboriginal (chronological age)	0.82 (0.76 to 0.89)	<0.0001
	Aboriginal (life expectancy)	0.85 (0.79 to 0.90)	<0.0001

Cessation of payments at age 65 precludes estimation of life expectancy for the welfare group.

## Results

**Figure 2:**
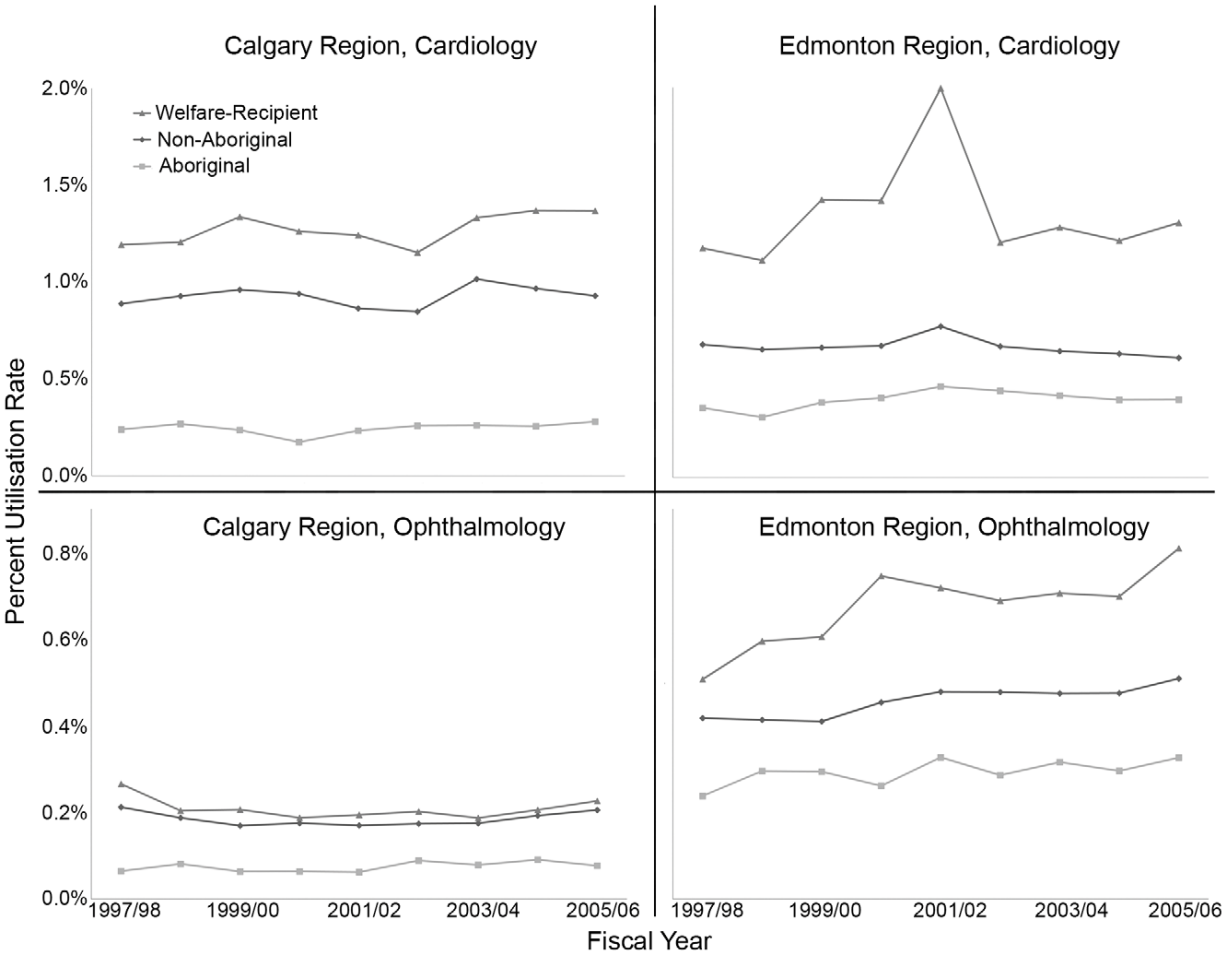
Regional cardiac and ophthalmic utilisation rates by population group and fiscal year. The population ranges are as follows: Aboriginal, 77 000–97 000; non-Aboriginal, 1.49–1.72 million; Welfare-recipient, 52 000–61 000.

Figure 2 shows the variation in healthcare utilisation rates, and during this period (1997 and 2006) the ranges are: (i) Cardiology Calgary: Welfare 1.15–1.37%, non-Aboriginal 0.85–1.02%, Aboriginal 0.18–0.28% (ii) Ophthalmology Edmonton: Welfare 0.51–0.81%, non-Aboriginal 0.41–0.51%, Aboriginal 0.24–0.33%. The Welfare group utilised healthcare at the highest rate, followed by the non-Aboriginal group, whilst the Aboriginal group exhibited the lowest rate over all nine years studied. These inter-group differences did not vary by provider (Calgary, Edmonton) or subspecialty (Cardiology, Ophthalmology), and the statistically significant differences in the three groups' utilisation rates remain relatively constant over time. Average utilisation rates were next calculated for individual population groups based on the distance between residential post codes and each regional centre (Edmonton or Calgary). Utilisation rates dropped steeply with distance and did not vary significantly between the three groups (p = 0.233; Figure S1). Examination of utilisation rates over time, within each urban population centre, revealed comparable trends to the province-wide data (Figure S2, Table S2).

There were marked differences in the age distributions of the Aboriginal and non-Aboriginal populations as shown in Figure 1. Notably, just 2.7% of the Province's Aboriginal population are 65 years of age or older, compared with 10.7% of non-Aboriginals (Table S3). Accordingly, healthcare utilisation rates were calculated with adjustment for fiscal year and either chronological age or estimated life expectancy, with the results given in Table 1. Standardising for age shows that welfare recipients are more likely to utilise healthcare for both Cardiology and Ophthalmology than the general population. For a given age, Aboriginals have the same Cardiology utilisation rate, IRR: 0.97 (0.89 to 1.04) as the general population. However, compared to the general population 5 years older (but with similar life expectancy), Aboriginals were less likely to utilise Cardiology care, IRR: 0.66 (0.62 to 0.71). Aboriginals had a lower Ophthalmology utilisation rate, IRR: 0.82 (0.76 to 0.89), and this was essentially unchanged if we compared the rate in the general population with that for a population of Aboriginals who were 5 years younger (Table 1). Lastly, Poisson regression analysis of cardiac and ophthalmic utilisation provided evidence of a significant interaction between age and Aboriginal status in patients over the age of 60 years [Cardiology, IRR: 0.66 (0.60 to 0.74); Ophthalmology, IRR: 0.78 (0.71 to 0.85), p<0.0001 (Table S1)].

## Discussion

Aboriginal populations have been demonstrated in multiple countries to have higher morbidity than non-native populations, encompassing both multi-system disorders (e.g. diabetes mellitus) and specific diseases. For instance, indigenous people in Australia and New Zealand experience higher rates of coronary artery disease [36], rheumatic heart disease [37], end stage kidney disease [38]. Furthermore, Aboriginal patients with type 2 diabetes are more likely to have microvascular complications and peripheral vascular disease [39]. Similarly, American Indians and Alaskan Natives have higher rates of heart disease and stroke [40], [41], diabetes [42] and diabetic retinopathy [28]. These findings accord with Canadian data revealing higher prevalences of cardiovascular disease [43], heart failure [44], and diabetes [26]. While some progress has been made towards addressing these disparities [12], [45], [46], profound differences remain between the health status of Aboriginal and non-Aboriginal people.

The precise reasons for this disparity remain unclear, but suggested causes include socio-economic inequality such as lower education and employment [4]–[5], [8]–[15], geographic isolation from healthcare facilities [3], [5], [11], as well as socio-cultural variation [4]–[5], [9], [16]–[18], particularly in terms of attitude towards seeking healthcare. For the Aboriginal population in the current study, displacement from ancestral lands to reserves frequently located in remote inhospitable regions may represent an important additional factor. From the multiple social determinants that contribute to health, including education and housing [21], we investigated healthcare utilisation because of its direct association with health status. Our study shows that significant disparities exist between Aboriginal and non-Aboriginal Albertan residents at the earliest stage of the treatment process, when they first accessed subspecialty (cardiac and ophthalmic) services. On an unadjusted basis, these differences were similar for Ophthalmology and Cardiology and also for Calgary and Edmonton, and remain evident even in comparison to economically disadvantaged individuals in receipt of welfare. As partly displayed in Figure S2, the markedly reduced rates at which large urban Aboriginal populations [from 2005/06, Edmonton (n = 21 930) and Calgary (n = 13 204)] utilise healthcare concur with our findings from across the province. They suggest that neither geographical isolation nor transportation explain the reduced rate at which Aboriginal residents utilise healthcare compared to non-Aboriginal Albertans.

The discrepant age distribution of Aboriginal and non-Aboriginal populations is of major importance both at individual and societal levels; and from the perspective of this study, in terms of the most appropriate way to analyse data. Since health care consumption increases disproportionately in the final years of life [47], major differences in life span [Aboriginal 70–76 years, non-Aboriginal 77–82 years in 2001] [19], [48]–[49] impact chronological age-based corrections, with the most pronounced effect anticipated in the elderly. This reflects the fact that age is merely a proxy for risk factors whose prevalence increases substantially as people become old, and thus a case-mix adjustment for age may mask real differences in healthcare usage [50]. This view is supported by the significant interaction noted between age and ethnicity (Table S1). Accordingly, since the majority of healthcare is consumed in the last decade of life [47], it may be more appropriate to compare groups of individuals with the same life expectancies rather than the same chronological ages. To address this possibility, in addition to standard age-based correction, a comparison based upon estimated life expectancy (time to death) was employed. These parallel analyses demonstrated comparable highly significant differences in healthcare utilisation in Ophthalmology. In Cardiology, similar findings were evident with a very conservative adjustment for life expectancy [Aboriginal: IRR 0.66 (0.62 to 0.71)], that were not apparent with an age-based correction [IRR: 0.97 (0.89 to 1.04)] (Table 1). We interpret these data as indicating that on both unadjusted and adjusted bases, Aboriginals utilise healthcare on average at a reduced rate compared to the general population, and that life expectancy based corrections are advantageous for disciplines where pathology contributes to mortality, as well as for populations where average life-span is reduced.

A fundamental strength of this study is that it is an evaluation of a large population over nearly a decade, enabling healthcare utilisation rates and trends to be calculated for precisely defined population groups. Reliance on treaty Aboriginal status - a very tightly defined criterion - further enhances the rigour of the results. Indeed the consistent disparity observed between Aboriginal and non-Aboriginal populations is biased towards being an under-estimate since only federally registered Aboriginals [30] were included, with substantial (but unquantifiable) numbers of non-federally registered Aboriginals present in the two other populations studied. Inclusion of an appreciable number of non-federally registered Aboriginals in the general population and welfare-recipient groups, would skew their respective rates, so reducing the true disparities in healthcare utilisation. Additionally, considerable confidence can be placed in these results due to the size of the populations studied [approximately two thirds of the 3.29 million provincial population in 2006] [22], the large geographical area involved [approximately the same size as France] and the highly statistically significant differences [p<0.0001] observed. As the most affluent Canadian province with the highest per capita provincial healthcare spending [51], and a per capita GDP exceeding that of the USA [52]–[53] it is probable that greater healthcare disparities exist in less affluent regions. In the context of substantially greater disease prevalence in Aboriginal populations (e.g. diabetes mellitus: 3–5 fold) [27], the reduced healthcare utilisation rates are especially stark.

Another key finding of this study is the essentially constant nature of these differences over a nine-year period, demonstrating that significant impediments to utilising healthcare persist. Since socioeconomic status is a potential barrier to health [4]–[5], [8]–[15], the utilisation rates of non-Aboriginal individuals receiving welfare were determined to provide a comparator group. Interestingly, this group utilised healthcare at a consistently higher rate than both Aboriginals and other Albertans, indicating that economic factors alone do not account for the reduced utilisation rates of Aboriginals. Since geographic remoteness has also been reported to contribute to lower healthcare utilisation rates [3], [5], [11], urban residents of Edmonton and Calgary were investigated to control for distance from major health centres (Figure S2). As no significant differences were observed between population groups with increasing distance from major subspecialty centres (Figure S1), geographic isolation is also not a major contributor to the discrepant utilisation rates observed. Taken together these findings suggest non-economic causes for the gap in healthcare utilisation rates.

Notwithstanding the strengths of this study both in terms of the cohort size and time period analyzed, there are a number of limitations that merit mention. One relates to the retrospective data collection that precluded prospective analysis of specific findings. A second reflects the concern providing researchers with healthcare data from very large populations [54], which are only increased when Aboriginal populations are included [55]. Accordingly, the data were anonymized, grouped by 5 year increment and geographic region, before being provided to us, preventing any identification of individuals. This also precluded subsequent adjustment by other patient characteristics, such as sex, and more rigorous age- and life-expectancy-adjusted analyses. Once this study's findings are published, we will seek access to the full dataset to determine if our hypothesis that similar disparities are present in other disciplines is correct, and to evaluate the contribution of additional patient factors.

Although identifying differences at a population level is important, translation of such findings to improved health is essential. In this context, one potential contributor to the discrepant utilisation rates that is more specific to the populations studied is the different approach to healthcare amongst these groups. In contrast to Western individual autonomy, a key element of Aboriginal health is integration of the family or community group into decision-making [56]. Consequently, conventional health professional teams may represent a barrier to Aboriginal healthcare utilisation. A second potential factor relates to the long history of maltreatment endured by these Aboriginal adults and children [4]–[5], [9]–[10], [57]–[60], resulting in a deep distrust of institutions and potentially profound effects on the likelihood that individuals seek voluntary treatment. If healthcare utilisation rates reflect the contributions of multiple factors, increasing the number of Aboriginal healthcare professionals coupled with increasing cultural sensitivity amongst non-Aboriginal health professionals may prove effective approaches to begin addressing the differences observed. Although it was possible to partially address the contributions of income (via welfare status) and geography, multiple additional socioeconomic factors merit consideration. For instance, Aboriginal peoples are over-represented amongst the homeless [61], with homelessness likely to hinder seeking healthcare [62]. Unfortunately, whilst such social determinants of health cannot be investigated through a population database study, they need to be addressed for disparities in health status to be solved [15]. In this context, our study's methodology for quantifying healthcare utilisation at local and regional levels offers the ability to move beyond defining the extent of the problem to start measuring the efficacy of changes introduced to address it. It may now become feasible, by assessing the effect of initiatives directly on healthcare utilisation rates, to determine the value of specific interventions for addressing the disproportionate burden of disease experienced by Aboriginal peoples.

## Supporting Information

Figure S1 **Average Calgary cardiac utilisation rates from 1996–2006, by distance from Calgary and population group.** (*p* = 0.2332)
(TIF)

Figure S2 **Cardiac utilisation rates by Calgary residents by demographic factor, fiscal year and population.** The population ranges are as follows: Aboriginal, 9000–13 000; non-Aboriginal, 80 000–95 000; Welfare-recipient, 19 000–24 000.
(TIF)

Table S1 **Poisson regression analysis of cardiac and ophthalmic healthcare utilisation.** This analysis demonstrated a significant interaction between chronological age and ethnic status. [IRR: ratio of the access rate for the Aboriginal population relative to that of the general population].
(DOCX)

Table S2 **Regional cardiac and ophthalmic utilisation rates by demographic factor and fiscal year.** The populations range as follows: Aboriginal, 77 000–97 000; non-Aboriginal, 1.49–1.72 million; Welfare, 52 000–61 000.
(DOCX)

Table S3 **Age distributions of Albertan Aboriginal and non-Aboriginal populations (1997–2006).**
(DOCX)
