# Supplementary figures and images for: Disparities in Healthcare Utilisation Rates for Aboriginal and Non-Aboriginal Albertan Residents, 1997–2006: A Population Database Study
Source: PLoS One. 2012 Nov 12;7(11):e48355. doi: 10.1371/journal.pone.0048355 (PMC3495946; doi:10.1371/journal.pone.0048355)

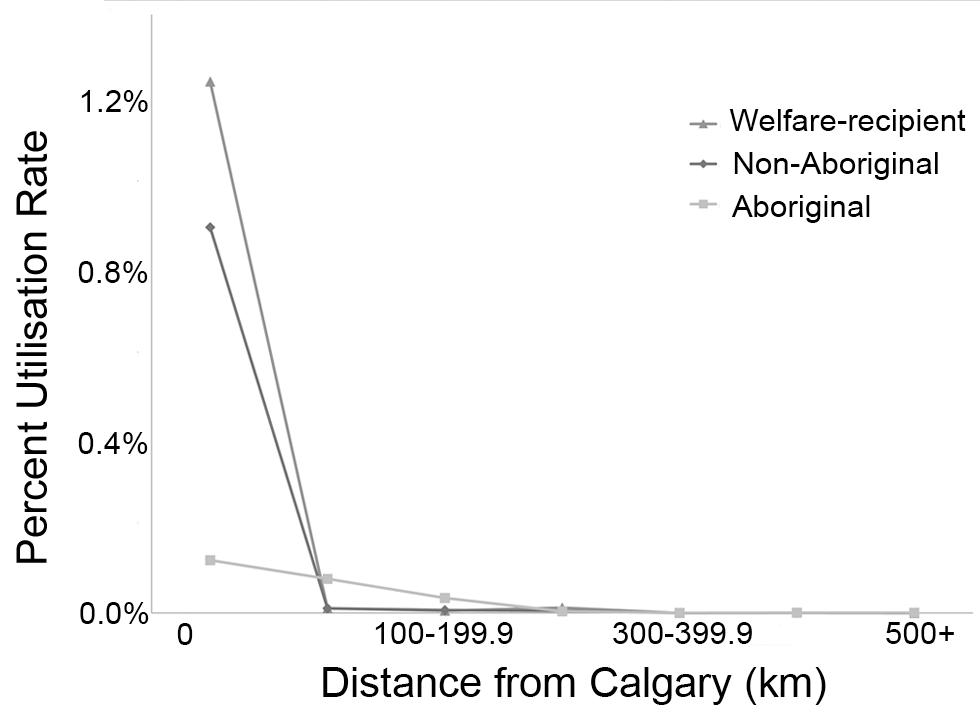

Supplement: Figure S1 — Average Calgary cardiac utilisation rates from 1996–2006, by distance from Calgary and population group. (p = 0.2332) (TIF) [file pone.0048355.s001.tif]

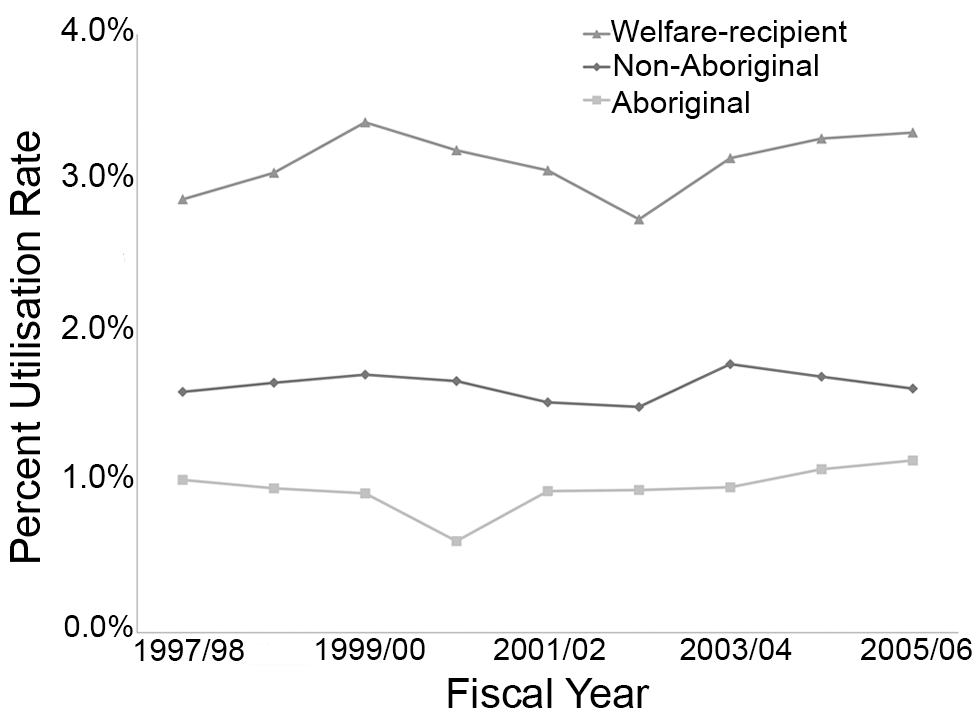

Supplement: Figure S2 — Cardiac utilisation rates by Calgary residents by demographic factor, fiscal year and population. The population ranges are as follows: Aboriginal, 9000–13 000; non-Aboriginal, 80 000–95 000; Welfare-recipient, 19 000–24 000. (TIF) [file pone.0048355.s002.tif]
