# Supplementary material for: Disparities in Healthcare Utilisation Rates for Aboriginal and Non-Aboriginal Albertan Residents, 1997–2006: A Population Database Study
Source: PLoS One. 2012 Nov 12;7(11):e48355. doi: 10.1371/journal.pone.0048355 (PMC3495946; doi:10.1371/journal.pone.0048355)
Supplement: Table S1 — Poisson regression analysis of cardiac and ophthalmic healthcare utilisation. This analysis demonstrated a significant interaction between chronological age and ethnic status. [IRR: ratio of the access rate for the Aboriginal population relative to that of the general population]. (DOCX) [file pone.0048355.s003.docx]

| **Subspecialty** | **Age Group** | **Incidence Rate Ratio**  (95% CI) | **p-value** |
| --- | --- | --- | --- |
| Cardiology | 0 - 29 | 0.99 (0.90 - 1.11) | 0.997 |
|  | 30 - 59 | 0.94 (0.88 - 0.99) | 0.048 |
|  | 60+ | 0.67 (0.60 - 0.74) | < 0.0001 |
|  |  |  |  |
| Ophthalmology | 0 - 29 | 0.61 (0.58 - 0.65) | < 0.0001 |
|  | 30 - 59 | 1.12 (1.03 - 1.22) | 0.009 |
|  | 60+ | 0.78 (0.71 - 0.85) | < 0.0001 |
