# Supplementary material for: Disparities in Healthcare Utilisation Rates for Aboriginal and Non-Aboriginal Albertan Residents, 1997–2006: A Population Database Study
Source: PLoS One. 2012 Nov 12;7(11):e48355. doi: 10.1371/journal.pone.0048355 (PMC3495946; doi:10.1371/journal.pone.0048355)
Supplement: Table S2 — Regional cardiac and ophthalmic utilisation rates by demographic factor and fiscal year. The populations range as follows: Aboriginal, 77 000–97 000; non-Aboriginal, 1.49–1.72 million; Welfare, 52 000–61 000. (DOCX) [file pone.0048355.s004.docx]

|  | **Non-Aboriginal** | **Aboriginal** | **Welfare** |
| --- | --- | --- | --- |
| **Calgary, Cardiology** | (%) | (%) | (%) |
| 1997/98 | 0.888 | 0.239 | 1.193 |
| 1998/99 | 0.928 | 0.268 | 1.206 |
| 1999/00 | 0.960 | 0.236 | 1.336 |
| 2000/01  2001/02 | 0.940  0.864 | 0.175  0.233 | 1.261  1.242 |
| 2002/03  2003/04  2004/05  2005/06 | 0.847  1.016  0.966  0.929 | 0.259  0.261  0.256  0.280 | 1.153  1.331  1.369  1.367 |
| **Edmonton, Cardiology** |  |  |  |
| 1997/98 | 0.682 | 0.357 | 1.176 |
| 1998/99 | 0.657 | 0.309 | 1.113 |
| 1999/00 | 0.666 | 0.385 | 1.424 |
| 2000/01  2001/02 | 0.675  0.775 | 0.409  0.467 | 1.420  1.996 |
| 2002/03  2003/04  2004/05  2005/06  **Calgary, Ophthalmology**  1997/98  1998/99  1999/00  2000/01  2001/02  2002/03  2003/04  2004/05  2005/06  **Edmonton, Ophthalmology**  1997/98  1998/99  1999/00  2000/01  2001/02  2002/03  2003/04  2004/05  2005/06 | 0.672  0.648  0.635  0.614  0.212  0.188  0.169  0.175  0.170  0.174  0.175  0.193  0.206  0.418  0.414  0.410  0.454  0.479  0.478  0.475  0.475  0.509 | 0.445  0.420  0.398  0.340  0.065  0.081  0.064  0.064  0.062  0.089  0.079  0.091  0.077  0.238  0.296  0.294  0.261  0.327  0.286  0.316  0.296  0.327 | 1.205  1.283  1.215  1.306  0.266  0.204  0.207  0.188  0.194  0.202  0.187  0.206  0.227  0.507  0.595  0.605  0.746  0.708  0.689  0.706  0.698  0.809 |
