# Supplementary material for: Disparities in Healthcare Utilisation Rates for Aboriginal and Non-Aboriginal Albertan Residents, 1997–2006: A Population Database Study
Source: PLoS One. 2012 Nov 12;7(11):e48355. doi: 10.1371/journal.pone.0048355 (PMC3495946; doi:10.1371/journal.pone.0048355)
Supplement: Table S3 — Age distributions of Albertan Aboriginal and non-Aboriginal populations (1997–2006). (DOCX) [file pone.0048355.s005.docx]

| Age  (years) | Proportion of Population (%)  Aboriginals Non-Aboriginals | |
| --- | --- | --- |
| 0-4 | 11.93 | 6.09 |
| 5-9 | 12.25 | 6.36 |
| 10-14 | 11.62 | 6.67 |
| 15-19 | 10.03 | 6.69 |
| 20-24 | 8.29 | 7.29 |
| 25-29 | 8.26 | 7.97 |
| 30-34 | 8.32 | 8.06 |
| 35-39 | 7.81 | 8.56 |
| 40-44 | 6.47 | 8.92 |
| 45-49 | 4.70 | 8.01 |
| 50-54 | 3.39 | 6.42 |
| 55-59 | 2.46 | 4.76 |
| 60-64 | 1.75 | 3.53 |
| 65-69 | 1.20 | 3.17 |
| 70-74 | 0.75 | 2.78 |
| 75-79 | 0.40 | 2.16 |
| 80-84 | 0.21 | 1.42 |
| 85-89 | 0.11 | 0.75 |
| 90+ | 0.05 | 0.37 |
| Total | 100 | 100 |
